# Effect of High-Humidity Hot Air Impingement Steaming on *Cistanche deserticola* Slices: Drying Characteristics, Weight Loss, Microstructure, Color, and Active Components

**DOI:** 10.3389/fnut.2022.824822

**Published:** 2022-04-26

**Authors:** Ziping Ai, Yawen Lin, Yongkang Xie, Samir Mowafy, Yue Zhang, Mengjia Li, Yanhong Liu

**Affiliations:** ^1^College of Engineering, China Agricultural University, Beijing, China; ^2^College of Food Science and Engineering, Bohai University, Jinzhou, China; ^3^Agricultural Products Processing Center, Henan Academy of Agricultural Sciences, Zhengzhou, China; ^4^Agricultural and Bio-Systems Engineering Department, Faculty of Agriculture, Alexandria University, Alexandria, Egypt

**Keywords:** *Cistanche deserticola* slices, high-humidity hot air impingement steaming, drying characteristics, color, microstructure, active components

## Abstract

*Cistanche deserticola* is one of the most precious herbal medicines and is widely used in the pharmaceutical and healthy food industries. Steaming is an important step prior to drying in the processing of *C. deserticola*. This research investigated the effects of high-humidity hot air impingement steaming (HHAIS) parameters such as temperature, time, and relative humidity (RH) on drying characteristics, weight loss, color, microstructure, and active components of *C. deserticola* slices. The results showed that the steaming process caused a weight loss in *C. deserticola*; however, increasing the RH reduced the weight loss. Starch gelatinization observed from the microstructure of the steamed samples explained their long drying time. The Page model can well fit the drying process with a high *R*^2^ (>0.956) under the drying conditions of 60°C and 6 m/s. Steaming increased the content of phenylethanoid glycosides, and the highest content was obtained at 95°C and 60% RH for 20 min, 75°C and 70% RH for 20 min, and 75°C and 60% RH for 30 min. The steamed samples appeared in an oil black color. When the color difference (Δ*E*) values were in the range of 16.79–20.12, the contents of echinacoside and acteoside reached the maximum. Steaming at 95°C and 60% RH for 20 min, 75°C and 70% RH for 20 min, and 75°C and 60% RH for 30 min are the optimum process conditions. The results from this work provide innovative steaming technology and suitable processing parameters for producing *C. deserticola* decoction pieces with a high quality, which will broaden its potential application in the functional health food industry.

## Introduction

*Cistanche deserticola* (known as “Rou Cong Rong” in China) is mainly found in the barren lands and deserts in some northern hemisphere regions, such as China, Iran, India, Mongolia, and so on (1–3). Dried *C. deserticola* slices have been commonly used as a tonic in China and Japan for many years to treat renal failure, impotence, female infertility, profuse metrorrhagia, and senile constipation (4). Modern pharmacological studies have also shown that the extracts of *C. deserticola* can enhance learning and memorization abilities, treat Alzheimer’s disease, improve immunity, exert antiaging and antifatigue effects, and promote bone formation (5). Additionally, the dried *C. deserticola* has been introduced to the healthy food market in the form of wine and tea obtained from the succulent stems (6). Phenylethanoid glycosides (PhGs) have been regarded as the major active components found in *C. deserticola*, among which echinacoside and acteoside are the representative markers for the quality control of *C. deserticola* in the Chinese Pharmacopoeia (7). In recent years, the supply of *C. deserticola* decoction pieces is far from being able to meet the consumer demands of the market due to its limited output and seasonality.

Due to the metabolic reactions, the post-harvested *C. deserticola* easily rots and deteriorates, degrading the active components (8). Fresh *C. deserticola* is usually blanched in hot water or steamed before the drying process to increase its active components content. Cai et al. (9) blanched *C. deserticola* slices at 70°C for 6 min in hot water before drying, which significantly improved the contents of echinacoside and acteoside compared with the directly sun-dried slices. Zou et al. (10) reported that a 10-min steaming followed by sequential freeze-drying obtained a better quality of *C. deserticola* compared with the direct freeze-drying. Peng et al. (6) investigated the effect of steaming time on bioactive components and antioxidant activity of *C. deserticola*, and their results suggested that steaming for 5–7 min significantly enhanced the total levels of the five PhGs and antioxidant activity in contrast to the direct oven drying. Peng et al. (11) observed that steaming promoted the accumulation of PhGs, polysaccharides, and soluble sugars in *C. deserticola* rhizomes and changed its color from yellow-brown to dark black. Additionally, our previous research also showed that the accumulation of active compounds during the processing of *C. deserticola* mainly occurred in the steaming stage (12). However, these studies mainly focused on the effect of steaming temperature and time on PhGs, and there is no report on the effect of relative humidity (RH) of the medium on the quality of *C. deserticola* during the steaming process.

Hot water blanching and steaming are the most common commercial steaming methods in the majority of the *Cistanche*-producing regions as they are simple, of low cost, and easy to implement. Otherwise, hot water blanching can lead to serious loss of soluble nutrients and wastage of water during the leaching process (13). Steaming can maintain water-soluble nutrients more than hot water blanching; however, the long steaming time often results in undesirable quality changes, especially at the low heat transfer and low steam velocity conditions (14). High-humidity hot air impingement steaming (HHAIS), a recently developed thermal treatment technology, gathers between the features of high-humidity hot air blanching and impingement technology (15). Compared with hot water blanching, HHAIS has the priority of sustaining the water-soluble nutrients and reducing the wasted water, where the product is processed in a high-humidity gas (15). Compared with steaming, HHAIS is more efficient as it has a higher heat transfer rate formed by the thin thermal boundary layer of the high-velocity high-humidity hot air (16). Due to these advantages, HHAIS has been applied to inactive enzymes and preserves the color of chili pepper (17), yam slices (18), and seedless grapes (16). Besides, HHAIS significantly enhances the drying rate of grapes and apricots by dissolving the wax layer on the grape surface and altering the cell ultrastructure and pectin nanostructure of apricots, respectively (14, 19). Xie et al. (20) mentioned that HHAIS can promote the conversion between gastrodin and *p*-hydroxy benzyl in *Gastrodia elata*.

During the HHAIS process, the governing factors of the steaming process are steaming temperature and time, and medium RH, which can affect the steaming performance, heat transfer efficiency, and products’ quality (21). Choosing an appropriate steaming temperature and time can reduce drying time, save energy, and improve product quality (22). To the best of our knowledge, no study is available concerning the effect of HHAIS temperature, time, and RH on the drying kinetics and physicochemical quality of herbal medicines, especially the changes of microstructure after steaming, as well as it is still unclear whether the drying time is related to the microstructure during the steaming process. Therefore, the objectives of this study are to (1) investigate the effects of steaming temperature, RH, and time on the drying characteristics and microstructure of *C. deserticola*, and the relationship between the drying characteristics and microstructure; (2) explore the effects of HHAIS conditions (e.g., temperature, RH, and time) on the physicochemical quality (weight loss, color, and PhGs’ content) of *C. deserticola*; and (3) examine the adequate mathematical model describing the drying process of *C. deserticola* slices under different HHAIS pretreatment. This work will provide essential information for selecting an optimal steaming technology and process parameters to achieve high-quality *C. deserticola* decoction pieces production.

## Materials and Methods

### Raw Materials and Sample Preparation

The fresh *C. deserticola* [*Cistanche tubulosa* (Schenk) Wight] were obtained from the Hetian region in Xinjiang Province, China. After the samples arrived at the laboratory, the inflorescence parts were removed with a ceramic knife. All the *C. deserticola* were stored in a ventilated area at room temperature before the experiments. To retain the consistency of the physical properties of the examined materials, the samples were collected regarding the similarity in size (average length, diameter, and weight were 14.12 ± 3.13 cm, 7.21 ± 1.25 cm, and 209.12 ± 5.89 g, respectively). Before the experiments, the surface of the raw sample was cleaned with clean water, and blotting papers were used to drain the excess water, then the surface’s skin was removed. Taking into account the variation in the content of PhGs along the longitudinal direction (from the bottom to the top) (2), each fresh *C. deserticola* rhizome was cut lengthwise into two halves (one-half formed the pretreatment samples and the other half formed the control samples) (20). Then, the two groups of samples were sliced into 5-mm thickness using an industrial food slicer. The initial moisture content of the samples was evaluated using a hot air oven at 105°C for 24 h according to the General Rule 0832 of Chinese Pharmacopoeia (7).

### High-Humidity Hot Air Impingement Steaming Pretreatment

**FIGURE 1:**
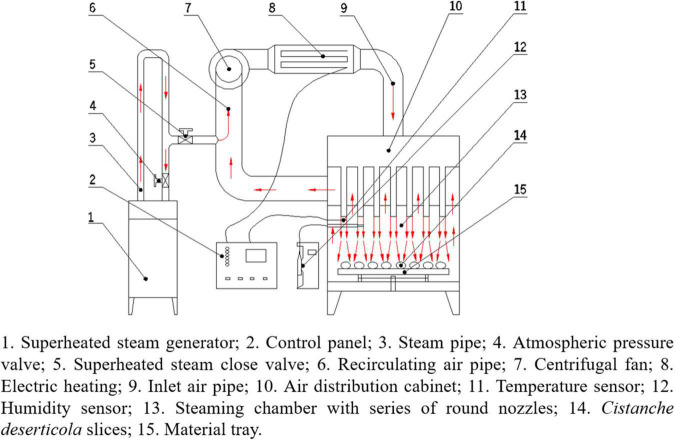
Schematic diagram of equipment used for high-humidity air impingement steaming (HHAIS) and hot air impingement drying (HAID).

The schematic diagram of the equipment used for HHAIS pretreatment and hot air impingement drying is shown in Figure 1. It was designed and developed by the laboratory of agricultural product processing technology and equipment, College of Engineering, China Agricultural University, Beijing, China. The device has been described in detail by Wang et al. (23). It involves a group of round inline nozzles, a steam generator (turned on during the blanching pretreatment and turned off during the drying process), an electric heater for heating the air, a centrifugal fan for supplying and circulating the airflow, and a proportional-integral-derivative governor (Omron, model E5CN, Tokyo, Japan) to dominate the steaming and drying temperature.

To explore the effects of steaming temperature, RH, and time on the drying characteristics and product quality of *C. deserticola* slices, single-factor experiments were designed as shown in Table 1 based on the pre-experimental results. The steaming process was conducted at four different temperatures (75^°^, 85^°^, 95^°^, and 105^°^C), four RH (50, 60, 70, and 80%), and four different steaming times (10, 20, 30, and 40 min) at a fixed air velocity of 14.0 ± 0.5 m/s. When the influence of one HHAIS parameter was studied, the other two parameters were kept constant and their values were chosen according to the study of Wang et al. (17) with some modifications based on the best matching between temperature, time, and RH for this material. When the temperature and RH reached the setting points, about 165 g of the samples were spread in a single layer on a stainless steel wire grid and put inside the steaming chamber and brought out when the steaming time was achieved. After steaming, the treated samples were cooled under ambient conditions at about 25^°^C. Unsteamed *C. deserticola* slices were considered as a control sample.

**TABLE 1:** Experimental design of HHAIS *Cistanche deserticola* slices.

No	Steaming temperature (°C)	Steaming time (min)	Relative humidity (%)	Enthalpy (J/g)	Drying Temperature (°C)	Air velocity (m/s)
1	75	20	60	555.22	60	6
2	85	20	60	949.32	60	6
3	95	20	60	1765.30	60	6
4	105	20	60	4322.15	60	6
5	75	10	60	555.22	60	6
6	75	30	60	555.22	60	6
7	75	40	60	555.22	60	6
8	75	20	50	456.22	60	6
9	75	20	70	663.87	60	6
10	75	20	80	784.27	60	6

### Hot Air Impingement Drying

After HHAIS pretreatment, both steamed and control samples were dried in the hot air impingement drier described above. The drying experiments were conducted at constant air velocity and temperature (6 m/s and 60°C, respectively), according to our preliminary experimental results (12). After stable drying conditions were achieved in the drier, the steamed and control samples were spread in a single layer and placed inside the drying chamber. Weight loss of the samples was tracked during the drying process at 1 h interval by removing the drying tray out of the drier and weighing it with an electronic balance (SP402, Ohaus Co., NJ, United States) with a sensitivity of ± 0.01 g. The entire weight recording operation was completed within 30 s to ensure the accuracy of the drying data. After achieving a final moisture content of 10.0% (on wet basis), the drying process was stopped. All the experiments were conducted in three replicates.

#### Drying Characteristics

The moisture ratio (*MR*) of *C. deserticola* was determined using Equation (1) (24):


(1) $$MR=\frac{M_{t}}{M_{0}}$$

where *M*_*t*_ is the moisture content (dry basis) of the samples at any time of drying, g/g; and *M*_0_ is the initial moisture contents (dry basis), g/g.

### Page Model Simulation of Drying Processes

The Page model compensates for some shortcomings of the Lewis model by modifying two empirical parameters (n and k) (25). The Page model has been widely used to study the drying process of agro-products and medicinal materials due to its high accuracy and simplicity (26–28). The model is expressed according to Equation (2):


(2) $$MR=\text{exp}\left(-kt^{n}\right)$$

where *k* and *n* are the constants of the model and *t* is the drying time (min). The coefficient of determination (*R*^2^), reduced chi-square (*λ^2^*), and root mean square error (*RMSE*) (Equations 3–5, respectively) were used between the predicated and experimental data to detect the goodness of the model. The higher *R*^2^ values, the lower λ^2^, and *RMSE* values, the goodness of fit.


(3) $$R^{2}=1-\frac{\sum_{i=1}^{N}{\left(MR_{exp,i}-MR_{pre,i}\right)}^{2}}{\sum_{i=1}^{N}{\left(MR_{exp,j}-MR_{pre,j}\right)}^{2}}$$

(4) $$\lambda^{2}=\frac{\sum_{i=1}^{N}{\left(MR_{exp,i}-MR_{pre,i}\right)}^{2}}{N-n_{0}}$$

(5) $$RMSE=\sqrt{\frac{\sum_{i=1}^{N}\left(MR_{exp,i}-MR_{pre,i}\right)}{N}}$$

where *MR_*exp*,i(j)_* and *MR*_*pre,i(j)*_ are the experimental and predicted MRs, respectively; N represents the number of constants; and n_0_ refers to the number of observations.

### Weight Loss Ratio

After steaming and cooling, the water on the surface of the samples was removed using absorbent paper, and the steamed samples were weighed again with an electronic balance. The weight loss ratio after HHAIS pretreatment was calculated as follows (Equation 6):


(6) $$Weightlossratio=\frac{W_{0}-W_{1}}{W_{0}}\times 100\%$$

where *W*_0_ is the initial weight of fresh samples before steaming (g) and *W*_1_ is the weight of the steamed samples (g).

### The Microstructure of Samples Under Scanning Electron Microscopy

The vertical cross-sectional microstructure of dried *C. deserticola* slices was observed using a scanning electron microscope (SU3500, Hitachi Ltd., Tokyo, Japan). The samples were cut into 3 mm × 3 mm × 2.5 mm and fixed onto a double-sided conductive adhesive tape. After sputtering with a layer of gold (10 nm) for 90 s, the samples were observed under a high-vacuum condition of 10 Pa and an accelerating voltage of 15 kV. The images were collected at 300× magnification.

### Determination of Color Appearance

The color of the steamed and unsteamed dried *C. deserticola* slices was determined using a computer vision system (CVS) located in the laboratory of agricultural product processing technology and equipment, College of Engineering, China Agricultural University, Beijing, China, which has been fully described by Li et al. (29). The CVS consists of an illumination chamber equipped with a fluorescent lamp (J&K Photoelectronic System Co., Ltd., Shanghai, China), an industrial camera (Aca250014-gc, Basler, Ahrensburg, Germany), and a computer equipped with MATLAB software (R2017b, The MathWorks, Inc., Natick, United States). The camera was placed vertically at a distance of 18 cm from the sample to acquire the image. The illumination intensity of the lamp was set to 110. The values of L* (whiteness/blackness), a* (redness/greenness), and b* (yellowness/blueness) were obtained by running the MATLAB program. The images were preprocessed, where binary images were segmented, mask images were created, and the images were transformed from RGB to Lab space. Color difference (Δ*E*) was calculated according to the following Equation (7):


(7) $$\Delta E=\sqrt{{\left(L^{*}-L_{0}^{*}\right)}^{2}+{\left(a^{*}-a_{0}^{*}\right)}^{2}+{\left(b^{*}-b_{0}^{*}\right)}^{2}}$$

where $L_{0}^{*}$, $a_{0}^{*}$, $b_{0}^{*}$ are the values of the control samples; L*, a*, b* are the values of the treated samples. Δ*E* is the color difference between the control and the treated samples. All measurements were carried out in triplicate.

### Determination of Echinacoside and Acteoside

The sample preparation of the reference solution and test solution followed the descriptions archived in the Chinese Pharmacopoeia Commission (7) with minor modifications. In brief, the sample powder (sifted through 65 mesh sieve) was accurately weighed 1 g and then extracted by ultrasonication with 50 ml 50% aqueous methanol solution for 40 min (250 W, 35 kHz). After using a centrifuge at 8050 × *g* for 10 min at 4°C, the supernatant was collected and filtered through a 0.22 μm membrane and stored at 4°C for further analysis.

Determination of echinacoside and acteoside from *C. deserticola* samples was accomplished by modifying the Ma et al. (30) method. The analysis was conducted using an Agilent 1260 series HPLC system consisting of a quaternary pump, degasser, autosampler, column oven, and diode array detector (Agilent Technologies, Santa Clara, CA, United States). The chromatographic separations were performed on an Agilent Zorbax SB-C18 column (250 mm × 4.6 mm, 5 μm) operated at 30°C. The detection level was adjusted to 330 nm wavelength. Solvent A (methanol) and solvent B (0.1% aqueous formic acid, v/v) formed the mobile phase, that flowed at a rate of 1 ml/min. The injection volume of the test solution was set as 10 μl.

Considering the great difference between different individuals of materials in this study, the change ratio (IF, %) was used to clarify the effect of HHAIS pretreatment on the content of active components in *C. deserticola*. The change ratio was calculated using the following Equation (8):


(8) IF=(S-F)/F×100%

where IF is the change ratio of the content of PhGs (%), S is the content of the PhGs of the steamed samples, and F is the content of the PhGs the unsteamed samples (control group). The positive IF value indicates an increase in the content of the PhGs, and the negative value indicates a decrease in the content of the PhGs after steaming.

### Data Processing and Statistical Analysis

All determinations were triple replicated, and the data are exhibited in means ± standard deviation. The statistical analysis was conducted by applying Duncan’s test and analysis of variance (ANOVA) using SPSS statistics software (version 21.0, SPSS IBM Corporation, Armonk, United states). Statistical significance for differences was examined at a probability level of 5% (*p <* 0.05).

## Results and Discussion

### Effect of High-Humidity Hot Air Impingement Steaming on Drying Characteristics

#### Steaming Temperature

**FIGURE 2:**
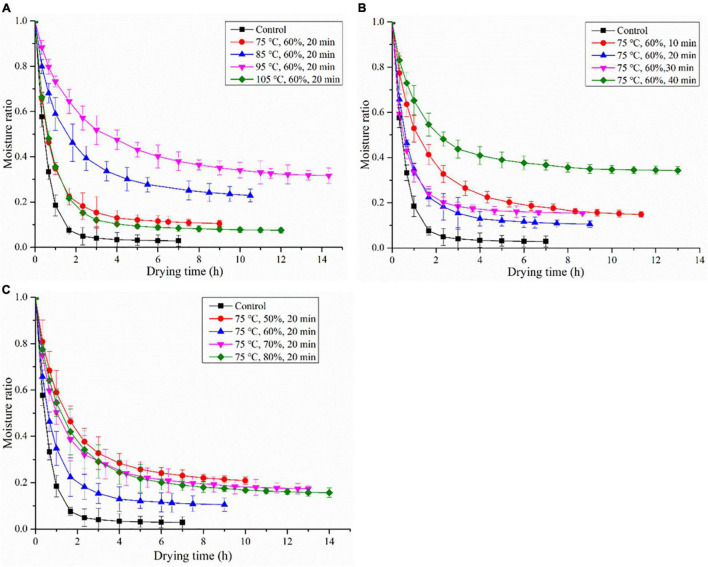
The drying kinetics of *Cistanche deserticola* under different HHAIS temperatures **(A)**, times **(B)**, and RHs **(C)**.

The influence of HHAIS temperature on the drying kinetics of *C. deserticola* was illustrated diagrammatically between *MR* vs. drying time. Figure 2A clarifies the effect of the steaming temperature on the drying time (the time consumed to reach the final moisture content of *C. deserticola*) at a constant steaming time and RH (20 min and 60%, respectively). The drying times were 9, 10.5, 14.5, and 12 h for the samples steamed at 75, 85, 95, and 105°C, respectively. In comparison with the untreated samples, the drying time of the steamed samples was prolonged by 29–107% with the rise of the steaming temperature from 75 to 105°C, indicating that the HHAIS pretreatment increased the drying time of *C. deserticola*. Interestingly, the current results are contrary to the previous studies on seedless grapes (16), in which the drying curves of grapes at different HHAIS temperatures almost overlapped. In general, due to the diversity of tissue properties and chemical compositions of different materials, the influence of steaming conditions on drying kinetics may also diverge. The phenomenon shown in this study may be due to the polysaccharide hydrolysis (content decreased from 16.78 to 12.34% after steaming) and starch gelatinization caused by high-temperature steaming pretreatment. This hypothesis has been confirmed in the steaming of *G. elata*, in which the monosaccharide and starch layer were observed to adhere to the cell surface of the steamed *G. elata* when its central temperature at the largest diameter was around 62.8–67.2°C, blocking the water migration channel of the porous material and resulting in a low drying rate (20).

#### Steaming Time

**FIGURE 3:**
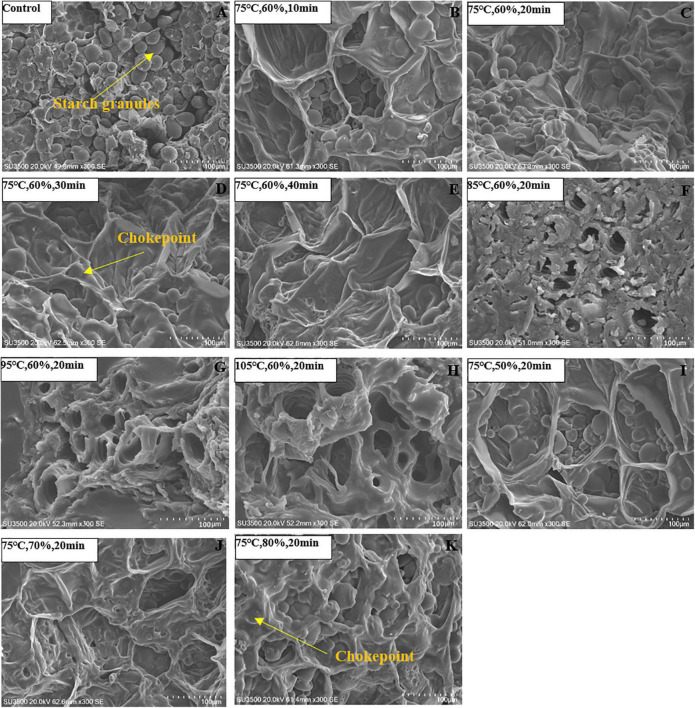
Vertical cross-sectional microstructures of *Cistanche deserticola* slices steamed under different temperatures, RHs, and times, respectively. Panel **(A)** represents the control group; Panels **(B–E)** represent the steaming time of 10, 20, 30, and 40 min, respectively; Panels **(F–H)** represent the steaming temperature of 85, 95, and 105 °C, respectively; Panels **(F–K)** represent the steaming RH of 50, 70, and 80%, respectively.

The effect of HHAIS time on the drying kinetics of *C. deserticola* under a constant steaming temperature of 75°C and RH of 60% is shown in Figure 2B. The drying times were 11.5, 9, 8.5, and 13 h at steaming times of 10, 20, 30, and 40 min, respectively. All the steamed samples had a longer drying time than the unsteamed samples. This phenomenon could be explained by the fact that, during the steaming process, the polysaccharide hydrolysis and starch gelatinization form a barrier layer that adheres to the cell surface, lowering the water migration rate. Similar results on the steaming of *G. elata* were published by Xie et al. (20), who found that the drying time required for *G. elata* increased by 187.5% when the HHAIS pretreatment time increased from 0 to 4.5 min. However, the drying time was reduced by 26% when the steaming time increased from 10 to 30 min, which was then extended when the steaming time rose to 40 min. The reason is that steaming can remove the air in the tissue, improve cell permeability, and reduce cell membrane resistance by modifying the tissue structure, which is conducive to water migration and diffusion (16, 23). For materials containing high sugar and starch, such as some herbal medicines, the negative effect of starch gelatinization may be greater than the positive effect of steaming on the drying rate when they are steamed longer enough for a high enough gelatinization degree (as shown in Figure 3) (18). This result can also be supported by the report from Chen et al. (31) that the blanched yam showed a higher moisture content than the unblanched ones at the same drying temperature, accompanied by the occurrence of starch gelatinization in the yam.

#### Relative Humidity

The effect of the steaming medium RH on the drying kinetics of *C. deserticola* at a fixed steaming temperature and time (75°C and 20 min, respectively) is presented in Figure 2C. The drying time decreased by 10% with the RH increased from 50 to 60%; however, the further increase in RH (from 60 to 80%) extended the drying time (from 9 to 14 h). This result showed that in the HHAIS pretreatment process choosing the appropriate medium RH is conducive to the migration of moisture during the drying process. The mechanism of the influence of medium RH on the drying kinetics of *C. deserticola* needs further studies.

### Page Model of *Cistanche deserticola* Drying

The calculated statistical parameters of the Page model for the drying of *C. deserticola* are presented in Table 2. As shown in the table, the Page model fits the experimental data with high *R*^2^ (0.956–0.993), low *RMSE* (2.18 × 10^–2^–4.29 × 10^–2^), and λ*^2^* (5.80 × 10^–4^–1.88 × 10^–3^) values, exhibiting suitability for predicting the change of moisture content of *C. deserticola* slices during the drying process after HHAIS pretreatment. The values of constant k firstly increased with the increase in HHAIS time and RH and then decreased, whereas the values decreased and then increased with the increase in steaming temperature, which showed that the k value can reflect the influence of HHAIS pretreatment on the drying kinetics of *C. deserticola*. The values of n firstly decreased with the rise of steaming temperature and RH and then increased; however, its values decreased with the increase in steaming time. To determine the values of the parameters k and n, polynomial equations were supposed to establish a set of equations between the model parameters and the HHAIS parameters, as shown in Equations 9–14. These equations have been validated in previous studies (28).


(9) $$\text{K}=a_{0}+a_{1}T+a_{2}T^{2}\left(R^{2}=0.947\right)$$

(10) $$\text{n}=a_{0}^{,}+a_{1}^{,}T+a_{2}^{,}T^{2}\left(R^{2}=0.998\right)$$

(11) $$\text{K}=a_{0}+a_{1}t+a_{2}t^{2}\left(R^{2}=0.981\right)$$

(12) $$\text{n}=a_{0}^{,}+a_{1}^{,}t\left(R^{2}=0.860\right)$$

(13) $$\text{K}=a_{0}+a_{1}\left(RH\right)+a_{2}{\left(RH\right)}^{2}+a_{3}{\left(RH\right)}^{3}\left(R^{2}=0.999\right)$$

(14) $$\text{n}=a_{0}^{,}+a_{1}^{,}\left(RH\right)+a_{2}^{,}{\left(RH\right)}^{2}+a_{3}^{,}{\left(RH\right)}^{3}\left(R^{2}=0.999\right)$$

**TABLE 2:** Page model statistical analysis results of *Cistanche deserticola* slices under different HHAIS conditions.

Steaming temperature (°)	Steaming time (min)	Relative humidity (%)	k (min)	n	R^2^	λ^2^	RMSE
75	20	60	0.964	0.520	0.977	1.88 × 10^–3^	3.99 × 10^–2^
85	20	60	0.524	0.499	0.981	1.27 × 10^–3^	3.27 × 10^–2^
95	20	60	0.338	0.507	0.981	8.92 × 10^–4^	2.82 × 10^–2^
105	20	60	0.974	0.552	0.972	2.08 × 10^–3^	4.29 × 10^–2^
75	10	60	0.621	0.537	0.983	1.24 × 10^–3^	3.28 × 10^–2^
75	30	60	1.003	0.399	0.975	1.77 × 10^–3^	3.84 × 10^–2^
75	40	60	0.451	0.394	0.956	1.84 × 10^–3^	4.03 × 10^–2^
75	20	50	0.538	0.540	0.977	1.58 × 10^–3^	3.68 × 10^–2^
75	20	70	0.672	0.436	0.973	1.64 × 10^–3^	3.81 × 10^–2^
75	20	80	0.622	0.491	0.975	1.65 × 10^–3^	3.83 × 10^–2^
Control			1.583	0.923	0.993	5.80 × 10^–4^	2.18 × 10^–2^

Accordingly, Equations 15–17 were developed to predict the moisture change during the drying process of *C. deserticola* treated by different HHAIS processes.


(15) $$\text{MR}=\text{exp}[\left(-22.29+0.4858T-0.00269T^{2}\right)t^{1.742-0.02866T+1.65\times 10^{-4}T^{2}}]$$

(16) $$\text{MR}=\text{exp}\left[\left(0.2413-0.1072t+0.002238t\right)t^{-0.0055t+0.6t}\right]$$

(17) $$\text{MR}=\text{exp}[(45.96-2.15\left(RH\right)+0.03239{\left(RH\right)}^{2}-0.00016{\left(RH\right)}^{3})t^{-7.425+0.3952\left(RH\right)-0.00641{\left(RH\right)}^{2}+3.38\times 10^{-5}{\left(RH\right)}^{3}}]$$

### Effect of High-Humidity Hot Air Impingement Steaming on Microstructure

The vertical cross-sectional microstructures of dried *C. deserticola* slices under different steaming conditions are listed in Figure 3. It can be seen that the unsteamed sample was observed to have a large number of starch granules with a regular arrangement. After HHAIS pretreatment, the number and distribution of starch granules and tissue structure were severely changed. According to Figures 3A–H, fewer starch granules were observed with increasing steaming temperature and time when RH was maintained at 60%. The starch granules completely disappeared, forming a dense barrier layer that adhered to the cell surface when the steaming temperature and time reached 85°C and 30 min, respectively. Shen et al. (32) reported a similar conclusion about the microstructure changes of germinated brown rice during microwave drying and revealed that the dense barrier layer was caused by starch gelatinization. Therefore, we can infer that the abovementioned changes in the microstructure of *C. deserticola* slices were due to the starch gelatinization during the steaming process. Previous studies have also shown that higher steaming temperature and longer time will result in a higher degree of starch gelatinization (18). Furthermore, the collapsed organizational structure without voids or fissures (Figures 3E,H) increased the resistance to water migration, which further explains the results of the characteristics in section “Effect of High-Humidity Hot Air Impingement Steaming on Drying Characteristic. Figures 3I–K show the microstructure of the dried *C. deserticola* slices at different steaming RHs, where a larger number of starch granules can be found on the cell surface at lower RHs (50–60%). When RH reached 70%, some chokepoints appeared on the cell surface due to starch gelatinization and thermal degradation of polysaccharides, which may explain the phenomenon that it was not conducive to drying when the RH value was too high.

### Effect of High-Humidity Hot Air Impingement Steaming on Weight Loss Ratio

**FIGURE 4:**
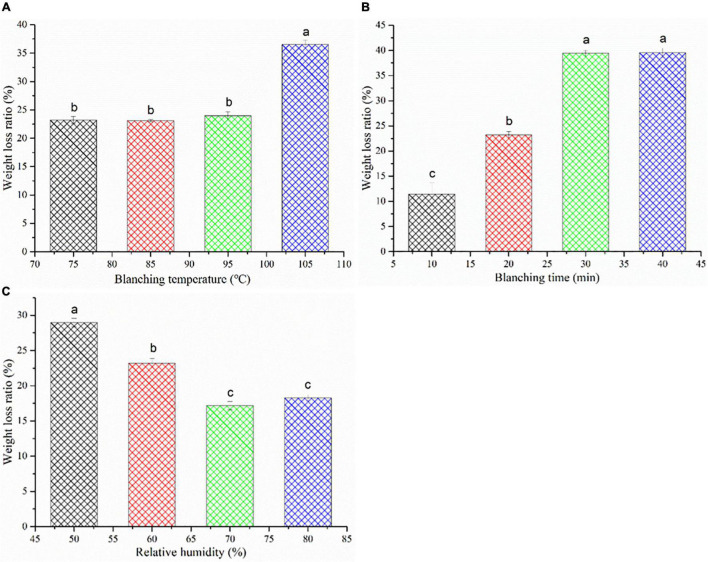
Weight loss ratio of *Cistanche deserticola* under different HHAIS temperatures **(A)**, times **(B)**, and RHs **(C)**. Different letters on data bars within each tested parameter indicate a significant difference (*p* < 0.05).

Usually, the weight of *C. deserticola* slices directly determines its commercial value in the medicinal herb market (6). Weight loss is a serious problem in the processing of agricultural products, which not only reduces the economic value of the product but also has some negative effects on the product quality such as bad color, severe shrinkage, and poor texture properties (33). The effects of HHAIS on the weight loss ratio of *C. deserticola* at different temperatures, times, and RHs are presented in Figure 4. The weight loss increased from 23.20 to 36.52%, and from 11.43 to 39.55% with the increase in temperature and time, respectively. Among the steaming temperatures, steaming at 105°C showed a significant increase in weight loss ratio (Figure 4A), which may be attributed to the thin dried layer on the surface of *C. deserticola* samples formed by evaporating the water from the surface layer during the steaming process. Figure 4B illustrates the negative impact of the steaming time on the weight loss ratio. It has been reported that the weight loss is closely related to the structure of the sample, and the destruction of the structure can reduce the capability of capillaries for holding the water (34). Therefore, the increase in the weight loss ratio may be due to the collapse and folding of the cell wall during the prolonged steaming, which leads to the destruction of the cell structure. This hypothesis can be proved by the microstructure diagram in Figure 3. In addition, the leakage of water-soluble nutrients such as polysaccharides, soluble sugars, and PhGs during the steaming process can also cause weight loss. This hypothesis was also supported by the report from Peng et al. (6) that the color of wastewater collected after steaming was light yellow due to the presence of water-soluble nutrients.

As shown in Figure 4C, increasing the steaming medium RH had a positive effect on the weight loss ratio. The weight loss ratio decreased from 28.97 to 18.26% by increasing RH from 50 to 80%, reflecting the beneficial effect of rising RH on reducing the weight loss. This may be due to less water evaporation out of the sample under the high RH conditions. A similar phenomenon was observed by Sotome et al. (35), who found that the weight loss can be reduced by a hot water spray (as a highly humid medium) blanching system.

### Effect of High-Humidity Hot Air Impingement Steaming on Echinacoside and Acteoside

The effect of different HHAIS process parameters on the content of PhGs is shown in Table 3. The steamed samples had a higher content of PhGs compared to the unsteamed ones. From this study, it can be deduced that steaming at 95°C and 60% RH for 20 min, 75°C and 70% RH for 20 min, and 75°C and 60% RH for 30 min followed by drying at 60°C and 6 m/s are the optimum process conditions with the larger increasing ratio of the content of PhGs compared to the other conditions according to the ANOVA results in Table 3.

**TABLE 3:** Changes of bioactive component contents in *Cistanche deserticola* samples subjected to HHAIS under different temperatures, times, and RHs.

Steaming temperature (°C)	Steaming time (min)	Relative humidity (%)	Group	Content of PhGs (%)			Increasing ratio of PhGs (fold)		
				Echinacoside	Acteoside	Echinacoside+ acteoside	Echinacoside	Acteoside	Echinacoside+ acteoside
75	20	60	Control	3.37	3.47	6.84	1.21 ± 0.08^d^	−0.02 ± 0.03^h^	0.58 ± 0.04^d^
			Blanching	7.45	3.39	10.84			
85	20	60	Control	2.07	1.12	3.19	2.45 ± 0.19^b^	1.29 ± 0.14^b^	2.04 ± 0.19^b^
			Blanching	7.14	2.56	9.70			
95	20	60	Control	2.52	1.47	3.99	3.93 ± 0.41^a^	1.88 ± 0.20^a^	3.07 ± 0.32^a^
			Blanching	12.43	4.23	16.66			
105	20	60	Control	9.15	3.99	13.14	0.66 ± 0.12^f^	−0.40 ± 0.04^g^	0.34 ± 0.03^e^
			Blanching	15.23	2.41	17.64			
75	10	60	Control	3.92	1.70	5.62	0.52 ± 0.08^f^	0.12 ± 0.02^f^	0.40 ± 0.04^e^
			Blanching	5.95	1.90	7.85			
75	30	60	Control	2.05	1.26	3.31	1.53 ± 0.12^c^	0.88 ± 0.07^c^	1.28 ± 0.23^c^
			Blanching	5.19	2.37	7.56			
75	40	60	Control	3.66	2.00	5.66	−0.37 ± 0.04^i^	−0.49 ± 0.06^g^	−0.41 ± 0.02^f^
			Blanching	2.30	1.02	3.32			
75	20	50	Control	1.83	0.80	2.63	0.40 ± 0.03^g^	0.29 ± 0.01^e^	0.37 ± 0.02^e^
			Blanching	2.56	1.03	3.59			
75	20	70	Control	3.31	1.32	4.63	0.95 ± 0.05^e^	1.18 ± 0.16^b^	1.02 ± 0.11^c^
			Blanching	6.47	2.88	9.35			
75	20	80	Control	2.48	1.53	4.01	0.23 ± 0.01^h^	0.44 ± 0.03^d^	0.33 ± 0.02^e^
			Blanching	3.04	2.21	5.25			

*Different letter indicates statistically significant difference at p < 0.05.*

#### Steaming Temperature

According to Table 3, the effect of steaming temperature on the content of PhGs was greater than that of steaming time and medium RH within the scope of each factor in this study, indicating that temperature was the dominant factor affecting the content of active components during the steaming process. When the steaming temperature increased from 75 to 95^°^C under a constant steaming time of 20 min and RH of 60%, the contents of PhGs, that is, echinacoside and acteoside, increased by 3.07 ± 0.32 times compared to 1.28 ± 0.23 and 1.02 ± 0.11 times for steaming time from 10 to 30 min (at a temperature of 75°C and an RH of 60%) and RH from 50 to 70% (at a temperature of 75^°^C and steaming time of 20 min), respectively. This result may be attributed to more inactivation of peroxidase and β-glucosidase by higher temperature during the steaming process that inhibited the degradation of PhGs, leading to higher levels of PhGs to be retained in the extracts (6). Moreover, exposing the fresh plants to the stress of high-temperature-high-moisture loss conditions may synthesize PhGs as phenolic compounds (36). However, when the steaming temperature increased from 95 to 105^°^C under a constant steaming time of 20 min and RH of 60%, the total increasing ratio of PhGs decreased from 3.07 to 0.34 times, demonstrating that steaming at 105^°^C for 20 min may lead to excessive steaming. Therefore, 95^°^C is recommended as the best HHAIS temperature to achieve the highest content of PhGs.

#### Steaming Time

As presented in Table 3, by increasing the steaming time from 10 to 30 min at a steaming temperature of 75°C and an RH of 60%, the contents of echinacoside and acteoside presented the same increasing trend and reached their highest values at 30 min. This result was inconsistent with the conclusion of Peng et al. (6), in which the content of PhGs increased with the increase in steaming time and reached the maximum at 93^°^C for 7 min. The inconsistency may be caused by the difference in blanching technology, slice thickness, and medium RH. Also, it has been shown that the heat treatment can cause a transformation in the chemical composition by destroying covalent bonds inside the material, thereby releasing more phytochemicals (37). However, HHAIS treatment for 30 min resulted in a highly extracted content of PhGs from *C. deserticola*, and when the steaming time exceeded 30 min, the detected contents of echinacoside and acteoside in the steamed samples were lower than those of the unsteamed samples. This was because the synthesis of PhGs in the HHAIS process was the result of the cumulative effect of temperature, time, and RH rather than the effect of a single factor. Oversteaming occurred at 75^°^C for 40 min, which caused degradation of the active components as shown in Table 3. Therefore, 30 min was recommended as the best HHAIS time. Similarly, in the study of onion blanching, Ren et al. (22) found that the negative impact of low temperature combined with long-term blanching (60^°^C for 3 min) on quality can be compensated by the combination of high-temperature and short-term blanching (70°C for 1 min).

#### Relative Humidity

At a fixed steaming temperature and time (75°C and 20 min, respectively), the effect of the steaming medium RH on the content of PhGs of *C. deserticola* is represented in Table 3. The accumulation of echinacoside and acteoside increased by increasing the RH from 50 to 70%, reflecting the beneficial effect of the higher medium RH on the content of PhGs. This result can be explained by several possibilities, namely, the combination of high temperature and high humidity can quickly inhibit the enzyme activity due to the rapid temperature rise inside the material (15), retaining the content of PhGs to a large extent. Besides, the combination of high temperature and RH, and a long time can increase the enthalpy relaxation of the material, expediting the chemical conversion (38). However, a lower increasing ratio of PhGs was found when RH increased to 80%. This phenomenon may be attributed to the fact that oversteaming occurred at an RH of 80%. What’s more, when the RH value was too high, too much saturated water vapor filled into the steaming chamber, causing part of the PhGs to dissolve in the blanching medium, leading to a decrease in the content of PhGs. Therefore, 70% RH was considered to be the best process parameter during HHAIS in *C. deserticola*.

### Effect of High-Humidity Hot Air Impingement Steaming on Color

**FIGURE 5:**
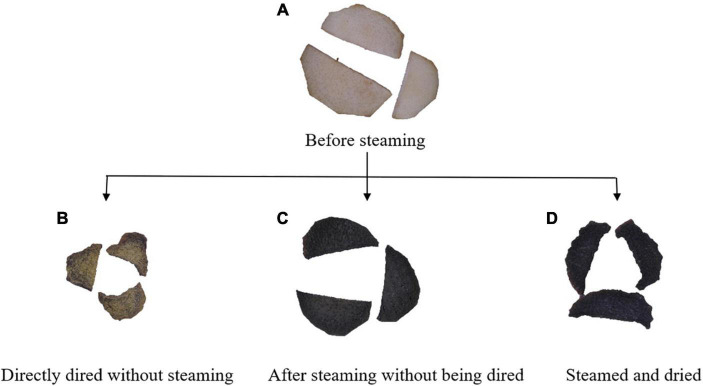
Digital photos of *Cistanche deserticola* slices processed at different states. **(A–D)** represent the samples before steaming, directly dried without steaming, after steaming without being dried, and steamed and dried, respectively.

The color of the dried products is one of the important parameters affecting their acceptability and trade value. The surface color parameters of dried *C. deserticola* slices were affected by different steaming conditions of HHAIS as shown in Table 4. Steaming temperature, RH, and time all affected the color of the dehydrated *C. deserticola* slices significantly (*p <* 0.05). The samples after steaming had lower L* and b* values compared with the control group. The digital photos indicated that the surface color of *C. deserticola* changed from yellow-brown to dark black after steaming (Figure 5). The steaming temperature had the greatest effect, where the high temperature decreased the values of L* and b* significantly (*p <* 0.05). The Maillard reaction, the main reaction responsible for the transformation of precursors into colorants, might exist during steaming, generating substances with a dark color (39). Similar results were observed for the dried *American Ginseng* (15) and the steamed *Polygonum multiflorum* (40).

**TABLE 4:** Effect of HHAIS temperature, RH, and time on the surface color parameters of *Cistanche deserticola*.

Steaming temperature (°C)	Steaming time (min)	Relative humidity (%)	L*	a*	b*	Δ E
75	20	60	29.40 ± 0.29^c^	4.05 ± 0.03^a^	5.44 ± 0.30^b^	13.99
85	20	60	29.74 ± 0.45^c^	3.50 ± 0.06^b^	5.09 ± 0.19^b^	13.66
95	20	60	27.02 ± 0.17^d^	4.14 ± 0.33^a^	3.03 ± 0.43^d^	16.79
105	20	60	23.10 ± 0.27^f^	1.52 ± 0.13^d^	−0.24 ± 0.27^f^	21.45
75	20	50	28.69 ± 0.38^d^	2.79 ± 0.26^bc^	2.66 ± 0.39^d^	15.21
75	20	60	29.40 ± 0.29^c^	4.05 ± 0.03^a^	5.44 ± 0.30^b^	13.99
75	20	70	23.87 ± 0.48^e^	2.52 ± 0.24^c^	1.63 ± 0.24^e^	20.12
75	20	80	25.71 ± 0.63^e^	3.27 ± 0.52^b^	2.97 ± 0.32^d^	18.00
75	10	60	34.42 ± 0.46^b^	3.64 ± 0.11^b^	6.72 ± 0.62^a^	8.86
75	20	60	29.40 ± 0.29^c^	4.05 ± 0.03^a^	5.44 ± 0.30^b^	13.99
75	30	60	24.65 ± 0.97^e^	1.97 ± 0.20^d^	1.74 ± 0.45^e^	19.34
75	40	60	27.91 ± 0.31^d^	4.15 ± 0.14^a^	3.87 ± 0.16^c^	15.74
Control			43.17 ± 0.52^a^	2.37 ± 0.09^c^	7.30 ± 0.18^a^	0

*Different letter indicates statistically significant difference at p < 0.05.*

The total color difference (△*E*), the total color difference between the steamed and unsteamed samples, can be observed by the naked eye when it is greater than 5 (15). The △*E* values increased from 13.99 to 21.45 as the steaming temperature increased. As steaming RH and time increased, △*E* values increased first then decreased. △*E* reached the maximum value at 30 min and 70% RH steaming condition. This phenomenon was consistent with the changing trend of PhGs, which indicated that there may be a correlation between the content of PhGs and △*E* values. Similar findings have been observed in the steam processing of *C. deserticola* by Peng et al. (41), who found that the brighter the *C. deserticola* powder, the higher the content of echinacoside; the yellower the powder, the higher the *polysaccharide* content. Peng et al. (11) also mentioned that *C. deserticola* has an oily black color and is beneficial to the steam blanching process.

## Conclusion

This study revealed the influence of HHAIS conditions (steaming temperature, RH, and time) on the drying and quality characteristics of *C. deserticola* slices. The results showed that the steamed samples need a drying time longer than the unsteamed samples and the increase in the steaming temperature, RH, and time prolonged the drying time. The Page model can well predict the change of moisture content during the drying process of *C. deserticola*. The microstructure showed that after HHAIS pretreatment a barrier layer was formed due to starch gelatinization and adhered to the cell surface, which explained the lower drying rate caused by steaming. In terms of product quality, the results showed that increasing the medium RH can effectively reduce weight loss during the steaming process. Steaming increased the contents of echinacoside and acteoside, and HHAIS conditions had a significant effect on the content of PhGs. Three steaming scenarios of 95°C and 60% RH for 20 min, 75°C and 70% RH for 20 min, and 75°C and 60% RH for 30 min had the highest echinacoside and acteoside content. The color analysis showed that the color appearance of *C. deserticola* changed from yellow-brown to dark black after steaming, corresponding to the decrease in L* and b* values, and the increase in Δ*E* values. When the Δ*E* values were in the range of 16.79–20.12, the content of echinacoside and acteoside reached the maximum. From this study, it can be deduced that steaming at 95°C and 60% RH for 20 min, 75°C and 70% RH for 20 min, and 75°C and 60% RH for 30 min followed by drying at 60°C and 6 m/s are the optimum process conditions as far as the increasing ratio of the content of PhGs is concerned. The findings in this research provide innovative steaming technology and optimal process parameters for producing *C. deserticola* decoction pieces. Regrettably, HHAIS has a limited loading capacity and the mechanism of RH’s influence on PhGs is still unclear. Further research is needed to clarify the mechanism of the effect of RH on the active components of *C. deserticola*. Additionally, the interaction of steaming temperature, time, and RH also needs to be analyzed through orthogonal optimization experiments.

## Data Availability Statement

The original contributions presented in the study are included in the article/supplementary material, further inquiries can be directed to the corresponding author/s.

## Author Contributions

ZA: conceptualization, methodology, date curation, writing-original draft preparation, software, and formal analysis. YWL: investigation and resources. YX and ML: methodology and software. SM: formal analysis investigation. YZ: investigation. YHL: resources, validation, funding acquisition, project administration, supervision, and writing-reviewing and editing. All authors contributed to the article and approved the submitted version.

## Conflict of Interest

The authors declare that the research was conducted in the absence of any commercial or financial relationships that could be construed as a potential conflict of interest.

## Publisher’s Note

All claims expressed in this article are solely those of the authors and do not necessarily represent those of their affiliated organizations, or those of the publisher, the editors and the reviewers. Any product that may be evaluated in this article, or claim that may be made by its manufacturer, is not guaranteed or endorsed by the publisher.
